# Annotations

**Published:** 1897-11-06

**Authors:** 


### ANNOTATIONS.

### Septic Infection in Operating Theatres.

Among the many matters which are involved in the dispute at the Western Infirmary, Glasgow, there is one which does but illustrate a cause of friction which, *in esse* or *in posse*, exists at many hospitals. viz., the conjoint use of the operating theatre. In old pre-antiseptic days the success of surgery at many hospitals was largely dependent on what went on within the theatre. It was there that all the cases, under all the surgeons, were subjected to one common influence, and, looking back from our present standpoint, we can understand how it happened that sometimes after months of successful work a wave of septic "accidents" would sweep through a whole hospital. The introduction of antiseptic surgery, however, altered all this, for a careful surgeon was able, by dint of strong chemical antiseptics, to make himself to a large extent independent of his surroundings. But many of the most cumbrous details of early Listerism were really only necessary in consequence of the all-pervading septicity which then prevailed. With increasing knowledge, and, it should be added, with increasing general cleanliness, simpler means have come into vogue, and the "aseptic" surgery of recent years has brought the old difficulty to the front again. So long as there is but one common operating-room, one set of instruments, one theatre sister, it is impossible for even the most careful surgeon to rise above the standard set by his colleagues. It is quite clear that to get the best work possible out of our hospitals each surgeon must have full responsibility, which cannot be the case when the most delicate part of his work has to be done under conditions over which he has no control. The difficulty is a grave one. Either we must have separate theatres, which is a very difficult matter, or an autocrat must be introduced—one who will prevent the theatre being misused.

### Nature's Cure for Over Population.

The plague in India continues to spread, and undoubtedly will give much trouble before it is got under. The disease has so marked a tendency to become endemic where the conditions of the soil are favourable to it, and the number of towns and villages in India which contain just that element of filth which is required for its local development is so great, that we can hardly venture to hope that it will quickly die out. We have in fact, both in regard to plague and famine, to face what is really a new difficulty in the history of such scourges. Our common humanity has led us to interfere with Nature's cure for the conditions by which they are produced—a savage and inhuman cure, but one that has again proved effectual. Over-population and a low standard of living, a willingness to live and to marry on what will only just keep body and soul together, are at the bottom of both plague and famine. A condition in which the sum total of a mans' labour is required to provide food and raiment leaves him no opportunity of putting by for a time of want, or of giving reasonable care to his sanitary surroundings. Nature's cure for over-population is pestilence and famine, and as far back as history reaches we find that by these means the balance between food and population has again and again been rectified, and accumulated wealth has been divided among a lessened population. We have chosen to interfere with Nature; the humane instincts which our religion has implanted in us have driven us to relieve famine, and, if possible, to hold pestilence in check, and yet, meanwhile, under the plea of not interfering with native religion and native customs, we have permitted the continuance of a condition of affairs which never could and never can go on without a periodical decimation. The problem before the ruling power is difficult, and in a sense dangerous, for its solution involves interfering with many of the prejudices and religious customs of the natives. But like other peoples, the natives cannot both "eat their cake and have it." If they are to enjoy the protection against Eastern plagues which Western civilisation tries to give them, they must in return be made to conform to Western standards of decent living. There must, then, be no hesitation in enforcing sanitary measures in India—unless, indeed, hardening our hearts and buttoning up our pockets, we let Nature take her course and exact her tribute in pestilence and famine.

### The Payment of Jurymen in Coroners' Courts.

At last there seems some hope that jurors in coroners' courts, so far as London is concerned, will receive some remuneration for their services, the Public Control Committee having reported to the London County Council in favour of two shillings per attendance being paid to all jurors, up to the number of 15, serving on any inquest, on condition that the jurors so serving shall be selected in proper rotation from the entire list of those liable to be called. There can be no doubt that this will greatly improve the position of the coroner's court, and will tend to remove that element of cantankerousness and of captious readiness to bring ridicule upon the proceedings by which so many unwilling jurymen revenge themselves for being forced to waste their time and lose their wages in the performance of a thankless public duty. The work of the coroner's court, if properly performed, is of the greatest importance, and there can be no doubt that, in an infinity of circumstances which cannot possibly be foreseen, the "crowner's quest" and the dread thereof afford a measure of protection to life which is given by no other agency at present in operation. Nothing can well be more unsatisfactory than the present condition of the law as to the registration of death. Perhaps the time will come when it will be impossible to bury a body without further inquiry merely because some ignorant old woman chooses to assert that the patient died of fits or measles, or some other cause of death which to the registrar seems not improbable; perhaps a time will come when a medical practitioner will no longer be forced by Act of Parliament, under a penalty of £20, to give a certificate of the cause of death in every case in which he has been in attendance during the last illness, however vague may be his knowledge and belief as to what the cause of death really was; and when such things happen the coroner's court will be restored to its rightful position as a court of inquiry, not only in cases of suspicion, but also in cases of doubt. If ever this should happen, then it will be still more necessary that the jurors on whom such important responsibilities will fall should receive some recompense for their labours.

